# Novel therapeutics for dry eye disease

**DOI:** 10.1080/07853890.2023.2189749

**Published:** 2023-05-15

**Authors:** Duoduo Wu, Louis Tong, Arun Prasath, Blanche Xiao Hong Lim, Dawn Ka-Ann Lim, Chris Hong Long Lim

**Affiliations:** a Department of Ophthalmology, National University Health System, Singapore, Singapore; b Singapore Eye Research Institute, Singapore, Singapore; c Duke-NUS Graduate Medical School, Singapore, Singapore; d Singapore National Eye Centre, Singapore, Singapore; e Singapore Cord Blood Bank, Singapore, Singapore; f Yong Loo Lin School of Medicine, National University of Singapore, Singapore, Singapore; g School of Optometry and Vision Science, University of New South Wales, Sydney, Australia

We read with interest the article by Sheppard et al. and congratulate the authors for a concise summary of therapies for Dry Eye Disease (DED) [1]. Given that DED is a complex, multifactorial disease with various subtypes; aqueous, lipid and mucin deficiencies, a broad understanding of available therapeutics is an important aspect of engaging and educating our patients. Treatment should also target the underlying pathogenic mechanism for maximal efficacy. Artificial tears for instance, have a varied composition to address various DED subtypes and severities. There are also other effective therapeutics used frequently in our practice that we would like to highlight to the readership.

Diquafosol sodium 3% (Diquas) produced a paradigm shift in DED treatment as a first-in-class mucin secretagogue. Although Diquas is a dinucleotide purinoreceptor P2Y2 receptor agonist designed primarily to improve surface wettability, it is effective across all types of DED. It improves lipid layer thickness, and is one of few therapies which reduces conjunctival epithelial damage and increases goblet cell density. It further inhibits apoptosis and inflammation [2]. Diquas has superior efficacy over artificial tear replacements in improving tear production, corneal fluorescein staining scores and tear film stability [3].

Autologous serum eye drops (ASED), platelet-rich plasma (PRP) and umbilical cord blood serum (UCBS) are blood derivatives effective in alleviating DED symptoms. These products contain properties that closely mimic the physiological tear film. Superiority of blood-derived products compared to artificial tears relate to the presence of epitheliotropic factors [4]. ASED and PRP improved patients’ symptoms, with PRP further improving visual acuities after one month of treatment [4]. PRP can be obtained from both peripheral and cord blood and has a shorter preparation time, with no incubation required. Cord blood PRP has higher levels of anti-inflammatory molecules over peripheral adult PRP [5]. UCBS treatment is associated with improvement in corneal epitheliopathy and nerve regeneration in patients with DED. It is purported to have superior efficacy over donor serum eye drops in reducing corneal damage and improving DED symptoms.

Other therapeutic options available in other centres but not mentioned in the article include topical rebamipide (Mucosta UD), topical perfluorohexyloctane, topical azithromycin, systemic pilocarpine, oral gamma-linolenic acid, amniotic membrane related treatment options; such as Prokera and amniotic membrane extract, topically administered mesenchymal stem cell-derived exosomes and scleral lenses such as PROSE® or EyePrintPRO™. Additional non-pharmacological devices targeting meibomian gland dysfunction not mentioned include TearCare®, iLux®, Mibo Thermoflo®. Promising therapeutics on the horizon include selenium sulfide containing ointments.

DED is reportedly the most prevalent ocular disease worldwide with significant impairment of vision-related quality of life. With information readily available to patients via the internet and an increasingly interconnected world, it is crucial that practitioners maintain a global view of available therapeutics. Although these options may not necessarily be readily available within each healthcare setting, this knowledge provides the basis of facilitating discussions with patients, maintaining patients’ confidence in the therapeutic relationship, and supports their ongoing evaluation and management.
